# Gender representation in leadership and speaking roles at rehabilitation medicine conferences in the UK: a 26-year analysis

**DOI:** 10.3389/fresc.2026.1744019

**Published:** 2026-05-19

**Authors:** Laura Edwards, Fattaneh Zonouzi, Phoebe Owen, Piera Santullo

**Affiliations:** 1 Centre for Rehabilitation and Ageing Research, University of Nottingham, Nottingham, United Kingdom; 2 Rehabilitation Medicine, University Hospitals of Derby and Burton NHS Foundation Trust, Derby, United Kingdom

**Keywords:** academic conference, diversity and inclusion, equity, gender representation, medical workforce, rehabilitation medicine

## Abstract

**Objective:**

To investigate gender representation in leadership and speaking roles at British Society for Physical and Rehabilitation Medicine conferences from 2000 to 2025 and compare participation with the national rehabilitation medicine workforce.

**Design:**

Retrospective cross-sectional analysis of conference programmes spanning 26 years.

**Setting:**

Annual scientific meetings of the British Society for Physical and Rehabilitation Medicine, United Kingdom.

**Participants:**

All individuals listed in conference programmes as participants with identifiable roles (session chair, invited lecturer, poster/platform presenter, panellist, other). Gender was determined using programme details, structured internet searches, or self-identification.

**Interventions:**

Not applicable.

**Results:**

Across 37 meetings, 1,214 participants were identified; gender was determined for 83%. Men held more roles overall (592 vs. 436; *p* < 0.0001), particularly as invited speakers (*p* = 0.0002). However, relative to Royal College of Physicians workforce benchmarks, women were significantly overrepresented across several roles. Representation fluctuated over time, with no consistent upward trend despite an increasing proportion of female Rehabilitation Medicine consultants.

**Conclusions:**

Men remain overrepresented in conference roles, but women participate at or above expected levels relative to their workforce share. Ongoing efforts are needed to address barriers and promote inclusive representation in Rehabilitation Medicine leadership and academic visibility across all dimensions of diversity, not only gender.

## Highlights

- Despite an increasing proportion of women in the UK rehabilitation medicine workforce, men remain overrepresented in active roles at national conferences, particularly as invited speakers.
- When adjusted for workforce demographics, women's participation in conference roles was proportionate or exceeded expectations, suggesting some progress in gender representation.
- However, representation has fluctuated over time without a sustained upward trend, indicating ongoing structural or cultural barriers to equity in academic visibility.
- Ensuring equity in conference participation requires more than proportional representation—it depends on meaningful inclusion in high-profile roles and leadership visibility.
- Organisers should implement inclusive policies, mentorship, and demographic monitoring to promote fair representation across gender and other dimensions of diversity.

## Introduction

Equality, diversity and inclusion are vital to health care (1) and scientific and medical research (2). A medical workforce that represents the diversity of the population served is crucial for providing high-quality care (3). Evidence demonstrates that patients achieve better outcomes when treated by diverse teams (1). Diversity in the medical workforce encompasses a range of characteristics including gender, race, sexuality, ethnicity, disability, socioeconomic background, and educational experience.

The composition of the UK medical workforce has changed substantially in recent years. Female trainees have outnumbered males for more than a decade (4) and in 2025, for the first time, women comprised the majority of doctors on the General Medical Council's national medical register (5). Despite this, significant gender disparities persist in senior and leadership clinical and academic roles. Women represent 41% of consultant physicians (6), 17% of consultant surgeons in England and Wales (7), 26% of General Practice partners (8) and 29% of medical directors (9). Similar inequities are seen in academic visibility, with women historically under-represented as invited speakers at professional conferences (10) or panellists (11) and are less than half as likely to achieve full professorship (12) compared to male colleagues.

Rehabilitation medicine (physical and rehabilitation medicine; physiatry) is often considered a specialty which offers a favourable work-life balance (13)—a feature sometimes perceived as signalling greater accessibility for women (14). The percentage of women consultants in rehabilitation medicine has been steadily increasing over the past decades, from 21% in 2004 (15) to 39% in 2022 (6).

Academic and professional conferences play a critical role in career development. They provide opportunities for disseminating research, building networks and enhancing visibility, all of which can influence promotion and leadership trajectories. In the UK, the British Society for Physical and Rehabilitation Medicine (formerly British Society for Rehabilitation Medicine) organises annual scientific meetings for professionals in rehabilitation medicine. The extent to which women or non-binary physicians are represented in visible roles such as session chairs or invited speakers at these events has not previously been examined. Understanding patterns of gender representation over time can help identify barriers, inform equality, diversity and inclusion initiatives, and promote equitable career opportunities in the specialty. To our knowledge, no previous study has examined gender representation in leadership and speaking roles at rehabilitation medicine conferences over such an extended period in the UK.

## Aims

This study aimed to characterise gender representation in visible academic and leadership roles at British Society for Physical and Rehabilitation Medicine (BSPRM) scientific meetings over a 26-year period. Specifically, we examined the proportions of men and women undertaking conference roles, assessed whether gender distribution differed across role types (session chair, invited lecturer, poster/platform presenter), and compared conference representation with the estimated gender distribution of the UK rehabilitation medicine workforce. We also explored changes in representation over time to identify potential trends relevant to equality, diversity and inclusion within the specialty.

These aims correspond to one primary outcome (overall gender representation) and three secondary outcomes (role-specific representation, comparison with workforce distribution, and temporal trends).

## Methods

### Study design and reporting guidelines

This was a retrospective cross-sectional study, analysing scientific meeting programmes from the British Society for Physical and Rehabilitation Medicine between 2000 and 2025. Although the study was conducted at a single time point, the dataset comprises repeated annual conference programmes, functioning as independent cross-sectional snapshots that allow descriptive examination of temporal trends.

Reporting follows the STROBE (Strengthening the Reporting of Observational Studies in Epidemiology) guidelines for observational studies. Conference programmes, including both standalone and joint meetings, were obtained from several complementary sources because no single complete archive exists for the full study period. Programmes were sourced from the Society's website (where available, and note that one year sourced thus was incomplete), from the first author's retained copies from previous attendance, and from a comprehensive personal archive maintained by a BSPRM member. All sources contained comparable core information (session titles, speaker names and roles), and no differences in structure or content were identified. The use of multiple sources ensured completeness of the dataset rather than introducing heterogeneity.

**Figure 1:**
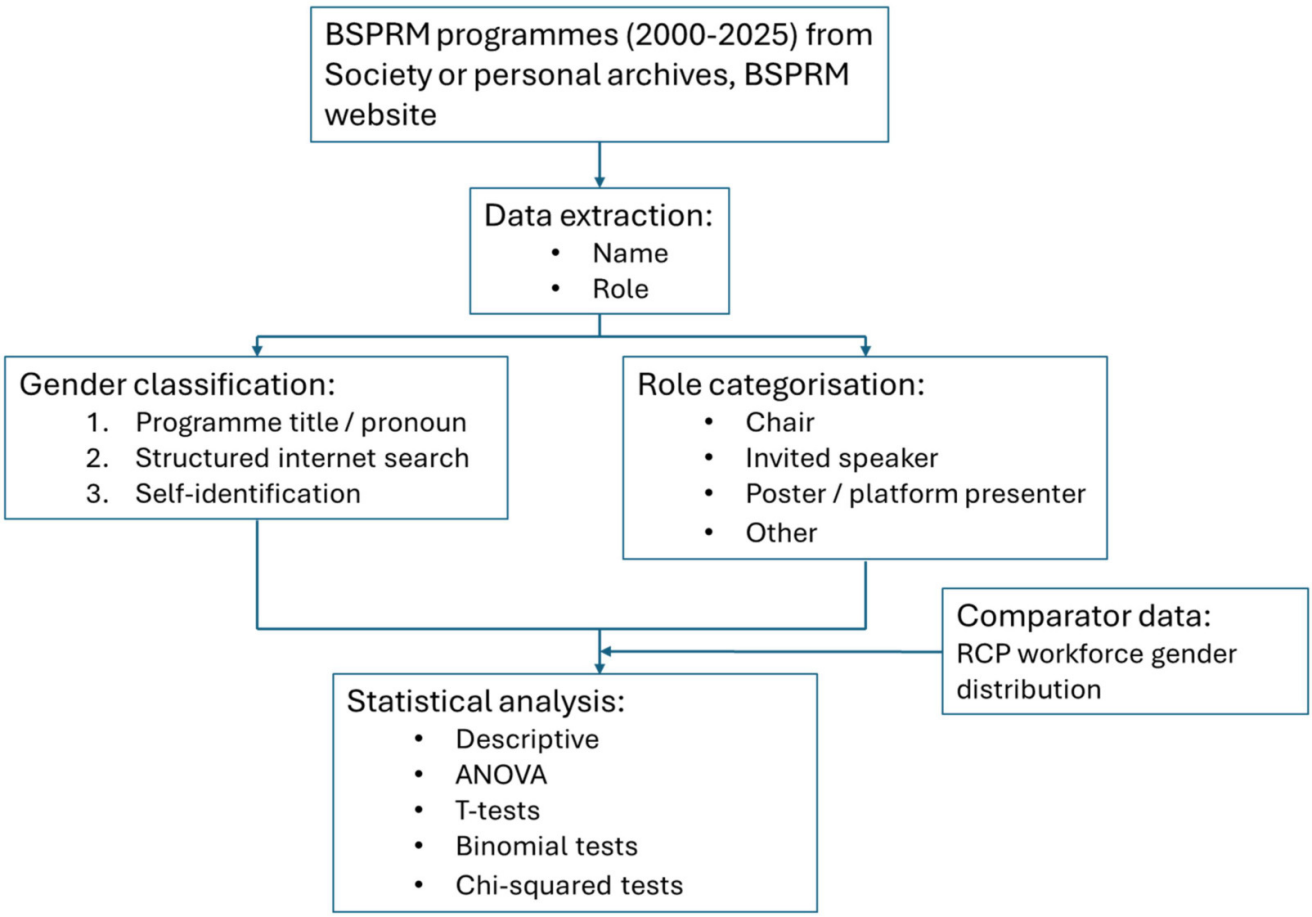
Methods flow diagram. BSPRM, British Society for Physical and Rehabilitation Medicine; RCP, Royal College of Physicians.

A flow diagram summarizing the methods is shown in Figure 1:

### Eligibility criteria

Individuals listed by name in conference programmes with an identifiable role.

### Outcomes and variables

All individuals listed as participants in each meeting were extracted into a database, along with their roles (e.g., session chair, invited lecturer, poster or platform presenter). The following variables were also extracted: meeting year, meeting type (BSPRM standalone vs. joint meeting), role category (chair, invited speaker, poster/ platform presenter, “other”—to include panellist, introductory speaker, welcome address and similar programme-listed roles not captured by the predefined categories.), presenter gender and workforce comparator proportions for corresponding year or epoch, based on available Royal College of Physicians (RCP) data.

The primary outcome was the proportion of roles held by men and women across all meetings.

Secondary outcomes included: (i) gender distribution within specific role categories, (ii) comparison with estimated gender distribution of the UK rehabilitation medicine workforce, and (iii) temporal trends in representation.

### Gender classification and quality control

Gender was determined from publicly available information using a three-stage process:

1. Programme information: gender-specific titles or pronouns provided in speaker biographies.
2. Structured internet search: where gender was not specified in the programme, a structured search (using combinations of name, specialty, talk topic, and institution where available) was undertaken. Up to four pages of search results were screened to identify authoritative sources (e.g., hospital or university profiles) specifying pronouns.
3. Self-identification: for one author of this paper without identifiable pronouns online, gender was self-reported.

No assumptions were made based on names or photographs. Individuals whose gender could not be identified through these routes were excluded from gender-specific analyses.

Approximately one-quarter of gender classifications were independently checked by a second researcher. Discrepancies were resolved through discussion and, if needed, review by a third researcher. All researchers are co-authors or collaborators on this paper.

#### Role categorisation

Roles were grouped into the following categories for analysis:

Chair—chairperson of session or lecture
Invited—keynote, plenary, or invited speaker
Poster/platform—presenter of submitted work selected for presentation (the first or only author named)
Other—roles such as member of discussion panel (panellist), opening remarks, announcements, introductions, or similar programme-listed contributions not captured by the predefined categories (not analysed further).

### Anonymisation

In some years, poster and platform abstracts were anonymised during peer review at the discretion of the organising committee. This anonymisation applied only to the review stage. All accepted presentations were published in the final programme with the presenting author's name listed. As anonymisation status could not be reliably determined for all years and did not affect the final dataset, it was not included as an analytical variable

### Time periods

For temporal analyses, meetings were grouped into five-year epochs (plus one six-year epoch for the earliest time period): 2000–2005, 2006–2010, 2011–2015, 2016–2020, 2021–2025.

### Comparator data

Comparator data on the gender distribution of UK rehabilitation medicine consultants (2000–2025) were provided by the Medical Workforce and Data Insight Team at the Royal College of Physicians (UK).

### Statistical analysis

Analyses were conducted using Microsoft Excel (Microsoft Corporation, Redmond, WA) and GraphPad Prism version 10.5 (GraphPad Software, San Diego, CA). Descriptive statistics summarised gender distribution by role and epoch. The unit of analysis was the role, not the individual. Individuals appearing multiple times across years or roles were counted separately for each appearance, reflecting the visibility and opportunity associated with each role.

ANOVA was used to compare proportions of identifiable genders across epochs; binomial two-tailed tests compared observed vs. expected gender counts based on either a theoretical 50:50 gender distribution of Royal College of Physicians workforce data.

Adjustments for multiple comparisons were applied as described in the results section.

### Ethics statement

As this study used publicly available information, no ethical approval was required.

## Results

Table 1 shows overview of results.

**Table 1:** Characteristics of participants identified in BSPRM conference programmes, 2000–2025.

Characteristic	Value
Total named participants (all roles, all years)	1,214
Gender identifiable, *n* (%)	1,028 (85%)
Gender not identifiable, *n* (%)	186 (15%)
Total number of meetings	37
Number of joint meetings (%)	9 (24%)
Meetings with complete programme data	36
Total roles analysed	1,028
Role distribution, *n*	
Chairs	205
Invited speakers	515
Poster/platform	137
Other	171
Participants excluded from gender-based analysis	186
Years with >90% gender identification	2010–2025

### Conference characteristics

Between 2000 and 2025, 37 British Society for Physical and Rehabilitation Medicine scientific meetings were held, with at least one meeting each year; 11 years hosted 2 meetings. Approximately one-quarter of meetings were joint events with other professional societies (full list in Supplementary File 1). Full programme details were available for 36 meetings. Information for 2023 was obtained from video recordings on the Society's website and may be incomplete.

### Participant identification

Across all meetings, 1,214 named participants were recorded. Some individuals appeared multiple times, reflecting participation across duplicate or different roles and conferences. Each role was counted separately. Gender could be determined for 1,028 participants (85%) and thus 186 participants were excluded from gender-based analysis. The proportion of participants with identifiable gender from paper or online sources increased from 53% in 2000 to over 90% from 2010 onwards (Supplementary File 2). The rate of identification was significantly lower in 2000–2005 compared with later epochs (ANOVA, *p* = 0.0036).

### Overall role distribution (2000–2025)

Over the study period, men held 592 roles and women held 436 roles. Compared to an expected equal distribution of roles, this is a statistically significant difference (2 tailed binomial *p* < 0.0001).

**Invited speakers**: men delivered significantly more invited talks than women (300 vs. 215 roles; *p* = 0.0002)
**Session chairs**: men chaired significantly more sessions [119 vs. 86 roles(*p* = 0.02)]
**Poster/platform presentations**: men had a greater number of poster/platform presentations (78 vs. 59 roles), but this did not reach statistical significance (*p* = 0.12)
**Other**: 171 participants held “other” roles across all the conferences, including panel participation, welcome talks, debates and commentaries. Due to the small numbers in each sub-category, this was not analysed further, but overall gender distribution in “other” was 95 men and 76 women.

**Figure 2:**
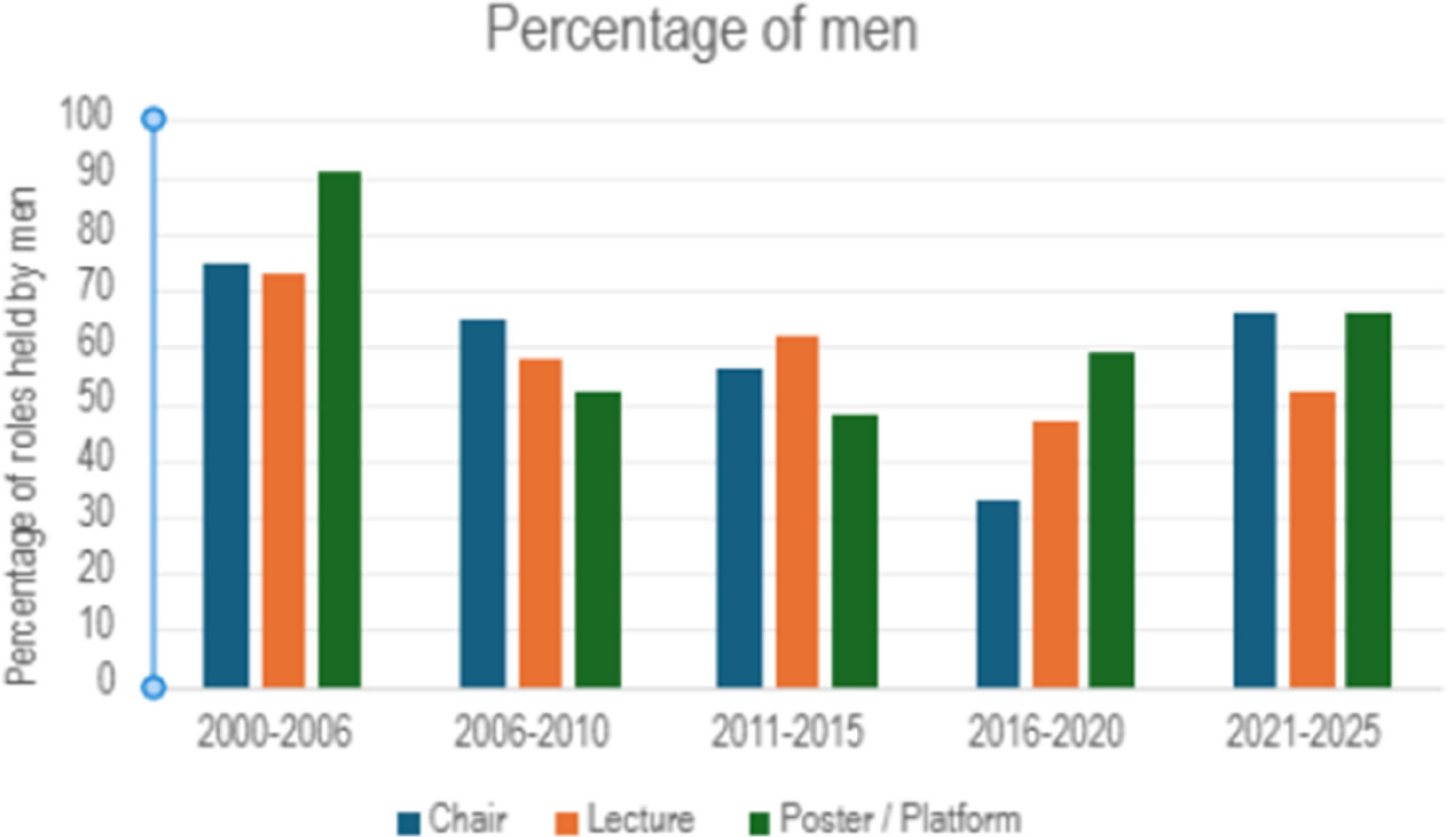
Percentage of roles held by men over time at British society for physical and rehabilitation medicine conferences.

At 12 of 15 time points, men held >50% of all roles (Figure 2).

### Comparison with workforce data

**Figure 3:**
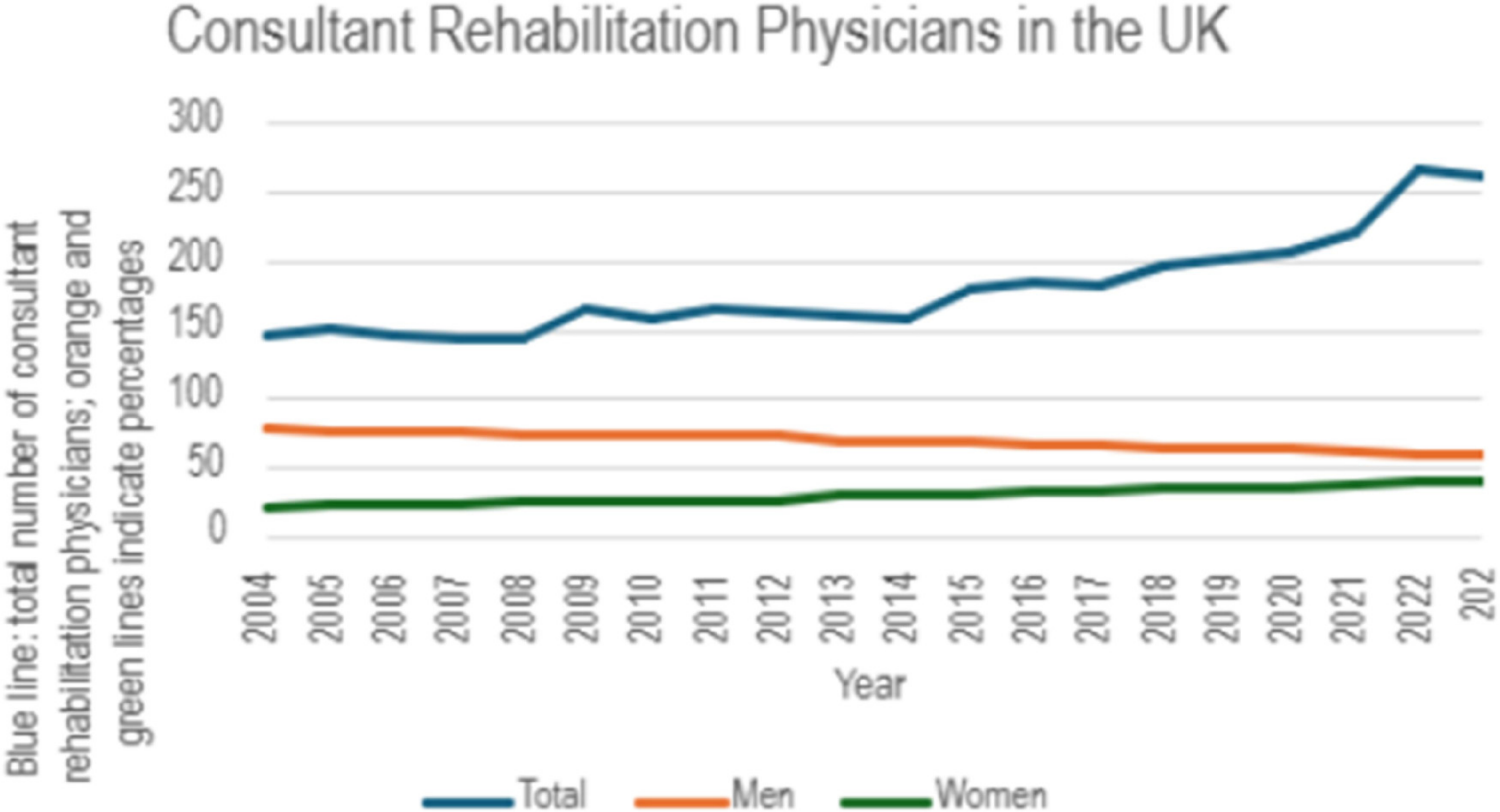
Percentage of consultant rehabilitation physicians in the UK by gender from 2004 to 2023.

According to Royal College of Physicians workforce data (available from 2004 onwards), the proportion of women rehabilitation medicine consultants increased from 21.1% in 2004 to 41.2% in 2022 (Figure 3; Additional File 3).

Comparisons of observed vs. expected proportions were adjusted for eight comparisons, giving a significance threshold of *p* < 0.00625. When compared with a theoretical 50:50 gender distribution, significantly more men overall participated in conferences than women (*p* < 0.0001) and gave invited talks (*p* = 0.0002) but there was no statistically significant difference between observed and expected proportions of chairs (*p* = 0.02) or poster/platform presentations (*p* = 0.12).

When compared with the Royal College of Physicians consultant workforce distribution (average of 70% men and 30% women), significantly more women were represented at BSPRM meetings across all roles: overall participation *p* < 0.0001; session chairs *p* = 0.0003; giving invited talks *p* < 0.0001; poster or platform presentations *p* = 0.0014.

### Changes over time

Binomial two-tailed tests compared observed men's representation rates to expected rates, based on either a 50:50 gender split and the Royal College workforce distribution for each epoch. Results are shown in Table 2 below:

**Table 2:** Comparing observed male conference participation to what would be expected from a 50:50 split (general population) and the UK rehabilitation medicine workforce over time.

Year	RCP data %age male consultants	%age men chairs	*P* vs. 50:50 split	*P* vs. RCP split	%age men invited (lecture)	*P* vs. 50:50 split	*P* vs. RCP split	%age men poster/platform	*P* vs. 50:50 split	*P* vs. RCP split
2000–2005	78	75	0.013	0.65	73	<0.0001	0.31	91	0.017	0.47
2006–2010	75	65	0.15	0.21	58	0.09	<0.0001	52	0.9	<0.0001
2011–2015	71	56	0.46	0.03	62	0.02	0.10	48	>0.99	0.01
2016–2020	66	33	0.03	<0.0001	47	0.52	<0.0001	59	0.52	0.50
2021–2025	60	66	0.01	0.43	52	0.69	0.13	66	0.21	0.79

After applying a correction for 30 comparisons (0.05/30 = significance threshold *p* < 0.00167), the following findings remained statistically significant:

1. An excess of men as chairs between 2000 and 2005 relative to a 50:50 gender split.
2. An excess of women as chairs between 2016 and 2020 relative to Royal College of Physicians data.
3. More invited women speakers during 2006–2010 and 2016–2020 compared to Royal College of Physicians data.
4. More women poster/platform presenters during 2006–2010 relative to Royal College of Physicians data.

## Discussion

Since 2000, significantly more men than women have held active roles in British Society of Physical and Rehabilitation Medicine conferences, with the disparity most pronounced among invited speakers. However, when benchmarked against the UK rehabilitation medicine consultant workforce, women have been proportionately well represented, and in some cases even over-represented in conference roles.

Across five to six year epochs, representation fluctuated, with higher female participation between 2006 and 2010. Despite the increasing proportion of women in the consultant workforce, there has been no consistent upward trend in conference participation over time. The overall male predominance mirrors findings from other medical and scientific conferences; however, incorporating workforce demographics provides important contextual nuance.

The relatively strong female participation in rehabilitation medicine conferences aligns with trends in other multidisciplinary, holistic specialties, such as geriatrics and palliative medicine, where women are better represented compared with procedure-intensive fields such as cardiology (6) or surgery (7). Reports suggest that newer specialties with multi-disciplinary, patient-centred cultures tend to diversify faster, while surgical fields remain among the slowest to achieve gender parity (16). These patterns may reflect a mix of historical cohort effects, differences in specialty attractiveness and career choices, and the influence of training and institutional policies.

Despite encouraging patterns, gender inequity persists in senior academic leadership in rehabilitation medicine in the UK. Of 14 full medically-qualified Professors of rehabilitation medicine in the UK between 2000 and 2025, only 3 are/were women (17). Similar disparities have been reported internationally. In the USA, a 2007 study found that 30% of male rehabilitation medicine academics held full professorship compared with 8% of women (18). More recent work has shown that women in rehabilitation medicine in the USA remain under-represented in high profile academic recognition, despite increasing representation in the workforce (19). Female researchers in rehabilitation medicine in the USA have reported lower levels of pay, fewer leadership roles and reduced rates of manuscript submission and grant applications compared with their male colleagues (18).

Rising numbers of female medical students and trainees may eventually translate into greater senior representation, but attrition remains a concern. In surgery, for example, the proportion of women falls from 54% in foundation training to 30% in senior training to just 12% at consultant level (20), illustrating the cumulative impact of structural barriers along the career pathway.

### Study limitations

This study provides a rare 26-year repeated cross-sectional review of the UK's main rehabilitation medicine conference, contextualized with national workforce data. Limitations include incomplete programme details for some early years, reliance on publicly available information for gender classification, and possibly misclassification. Workforce comparisons should also be interpreted with caution, as British Society for Physical and Rehabilitation Medicine events attract participants from other specialties and allied health professions, many of which have different gender distributions (e.g., physiotherapy and nursing are predominantly female (21, 22).

Poster and platform presentations rely on individuals submitting their work for selection (compared to participating as a chair, invited speaker or panel member for example) and so adds a further potential barrier of self-selection for voluntary submission. These are often reviewed by the selection committee anonymously or “blindly”, i.e., the committee is not aware of the name, gender or other identifying information of the submitting authors.

We focused solely on gender, and we recognize that this represents only one facet of equality, diversity and inclusion. Gender was chosen in part because it is typically easier to determine from public records and conference materials than other characteristics, a limitation acknowledged in other conference diversity analyses (23). Gender identification in this study relied in part on publicly available online information, which is influenced by individuals’ digital presence and the extent of detail provided on institutional or professional platforms. Searches were conducted broadly across web sources, without restriction to specific platforms, and visibility varied widely between participants. Female academic physicians have been shown to be less likely than male peers to achieve high online visibility, even when their offline scientific impact is similar (24). Individuals with less active or less detailed web presence were less likely to be identified in our study, so this digital visibility bias may have disproportionately affected women in our study.

### Suggestions for future practice and research

Although rehabilitation medicine conference participation appears relatively equitable, disparities in leadership and academic visibility persist. Targeted strategies could help sustain and build on progress, including mentorship and sponsorship programs tailored to women and underrepresented groups; family-friendly policies (e.g., childcare support and flexible scheduling), to remove barriers to participation, and systematic monitoring of demographic data at conferences to enable accountability and track progress.

Furthermore, inclusion policies and broader diversity initiatives must address other identities and intersectionality, recognizing the complex interactions between gender, ethnicity, disability, and sexual orientation.

Future research should extend beyond gender to examine other dimensions of equity, diversity, and inclusion, including ethnicity, socioeconomic status, disability, and LGBTQ+ identities. Investigating intersectionality will be crucial to understanding the layered barriers that affect recruitment to and participation and progression in rehabilitation medicine and other specialties. Qualitative work with underrepresented groups could illuminate cultural and structural challenges, while longitudinal cohort studies could clarify the long term impact of conferences participation on career trajectories and leadership attainment.

## Conclusions

In summary, men continue to predominate in UK rehabilitation medicine conference roles, particularly as invited speakers. However, relative to their proportion in the consultant workforce, women's participation is strong. Sustained efforts to promote equity and inclusion are required to ensure that future academic and clinical leaders in rehabilitation medicine reflect the diversity of the specialty and the population it serves.

## Data Availability

The raw data supporting the conclusions of this article will be made available by the authors, without undue reservation.

## Author's note

This paper was presented in poster form at BSPRM Annual Scientific Conference, September 2025.
