# Supplementary material for: Gender representation in leadership and speaking roles at rehabilitation medicine conferences in the UK: a 26-year analysis
Source: Front Rehabil Sci. 2026 May 19;7:1744019. doi: 10.3389/fresc.2026.1744019 (PMC13226536; doi:10.3389/fresc.2026.1744019)
Supplement: Supplementary file 1 [file Supplementaryfile1.pdf]

Supplementary table 1 – List of BSRM / BSPRM Scientific meetings, 2000-2025

Year	Meeting date and type
2000	July<br>November
2001	June<br>November
2002	May (joint with SRR)<br>November
2003	July<br>December
2004	April (1st day of 2 joint with Rehabilitation and Intermediate Care Nursing Forum)<br>September
2005	September
2006	July (joint with SRR)<br>November
2007	November
2008	May<br>October (joint with VRA)
2009	May<br>October

2010	September
2011	July<br><br>November
2012	June<br><br>November
2013	April (joint with VRA)<br><br>December
2014	October
2015	December
2016	November
2017	September
2018	October
2019	October (joint with SRR)
2020	November (joint with SRR and ACCPLD)
2021	November (join with SRR and ACCPLD)
2022	October
2023	July (joint with BASCIS) (programme taken<br><br>from videos available on BSPRM website)
2024	November
2025	September (preliminary programme taken<br><br>from BSPRM website)

Supplementary table 2- List by year of gender identification

Year	Total to identify	Found	Percentage found
2000	70	37	53
2001	81	60	74
2002	43	28	65
2003	12	10	83
2004	20	13	65
2005	15	14	93
2006	43	29	67
2007	40	34	85
2008	117	74	63
2009	67	60	94
2010	38	34	89
2011	63	57	90
2012	75	65	87
2013	26	26	100
2014	43	42	98
2015	26	24	92
2016	59	50	85
2017	43	38	88
2018	32	30	94

2019	56	56	100
2020	27	26	96
2021	63	57	90
2022	16	16	100
2023	26	26	100
2024	69	61	88
2025	44	43	98
