# Supplementary material for: Gender representation in leadership and speaking roles at rehabilitation medicine conferences in the UK: a 26-year analysis
Source: Front Rehabil Sci. 2026 May 19;7:1744019. doi: 10.3389/fresc.2026.1744019 (PMC13226536; doi:10.3389/fresc.2026.1744019)
Supplement: Supplementary file 3 [file Supplementaryfile3.pdf]

Supplementary information 3:

RCP data: The Medical Workforce and Data Insight Team at the Royal College of Physicians of the UK provided the following information regarding genders of rehabilitation medicine consultants (no data available before 2004):

2004 – total 147 – 21.1% female – 78.9% male

2005 – total 152 – 23.7% female – 76.3% male

2006 – total 147 – 23.8% female – 76.2% male

2007 – total 145 – 24.1% female – 75.9% male

2008 – total 144 – 25.0% female – 75.0% male

2009 – total 166 – 26.5% female – 73.5% male

2010 – total 158 – 25.3% female – 74.7% male

2011 – total 167 – 26.3% female – 73.7% male

2012 – total 164 – 25.6% female – 74.4% male

2013 – total 160 – 30.0% female – 70.0% male

2014 – total 159 – 31.4% female – 68.6% male

2015 – total 180 – 31.7% female – 68.3% male

2016 – total 186 – 31.7% female – 68.3% male

2017 – total 182 – 34.1% female – 65.9% male

2018 – total 197 – 33.5% female – 66.5% male

2019 – total 203 – 34.5% female – 65.5% male

2020 – total 207 – 34.8% female – 65.2% male

2021 – total 222 – 37.8% female – 62.2% male

2022 – total 267 - 41.2% female- 58.8% male.

2023 – total 263 - 40.3% female - 59.7% male
